# The SmpB C-terminal tail helps tmRNA to recognize and enter stalled ribosomes

**DOI:** 10.3389/fmicb.2014.00462

**Published:** 2014-09-02

**Authors:** Mickey R. Miller, Allen R. Buskirk

**Affiliations:** ^1^Nora Eccles Harrison Cardiovascular Research and Training Institute, University of Utah, Salt Lake City, UTUSA; ^2^Department of Molecular Biology and Genetics, Johns Hopkins University School of Medicine, Baltimore, MDUSA

**Keywords:** SmpB, tmRNA, decoding, ribosome stalling, EFTu

## Abstract

In bacteria, transfer-messenger RNA (tmRNA) and SmpB comprise the most common and effective system for rescuing stalled ribosomes. Ribosomes stall on mRNA transcripts lacking stop codons and are rescued as the defective mRNA is swapped for the tmRNA template in a process known as *trans*-translation. The tmRNA–SmpB complex is recruited to the ribosome independent of a codon–anticodon interaction. Given that the ribosome uses robust discriminatory mechanisms to select against non-cognate tRNAs during canonical decoding, it has been hard to explain how this can happen. Recent structural and biochemical studies show that SmpB licenses tmRNA entry through its interactions with the decoding center and mRNA channel. In particular, the C-terminal tail of SmpB promotes both EFTu activation and accommodation of tmRNA, the former through interactions with 16S rRNA nucleotide G530 and the latter through interactions with the mRNA channel downstream of the A site. Here we present a detailed model of the earliest steps in *trans*-translation, and in light of these mechanistic considerations, revisit the question of how tmRNA preferentially reacts with stalled, non-translating ribosomes.

## INTRODUCTION

Messenger RNA transcripts lacking stop codons pose a threat to the viability of all living organisms. Non-stop RNAs deplete the pool of available ribosomes because, unable to recruit release factors, they trap ribosomes for extended periods of time at their 3′-ends. They may also encode aberrant proteins with toxic activities. To address these issues, several mechanisms have evolved to detect and destroy non-stop RNAs as well as their aberrant protein products and to release and recycle stalled ribosomes. The non-stop decay (NSD) pathway first characterized in yeast, for example, shares similarities to other RNA quality control pathways and operates in many if not all eukaryotes (Shoemaker and Green, 2012).

In bacteria, the most common and effective system for rescuing stalled ribosomes consists of the universally conserved transfer-messenger RNA (tmRNA) and its protein partner, SmpB (Keiler et al., 1996; Karzai et al., 1999). tmRNA and SmpB are essential for growth in several species (Hutchison et al., 1999; Huang et al., 2000; Ramadoss et al., 2013b), are required for pathogenesis in others (Julio et al., 2000; Okan et al., 2006), and are a promising target for antibiotic development (Ramadoss et al., 2013a). Redundant mechanisms for releasing stalled ribosomes exist in a subset of bacteria. These include alternative release factors ArfA/YhdL, found in some proteobacteria (Chadani et al., 2010), and ArfB/YaeJ, found in many gram-negative bacteria (Chadani et al., 2011; Handa et al., 2011). However, the fact that tmRNA and SmpB are encoded in all sequenced bacterial genomes (Gueneau de Novoa and Williams, 2004) suggests that the alternative release factors are backup systems and that the predominant mechanism for dealing with non-stop mRNA in bacteria involves tmRNA.

tmRNA and SmpB recycle stalled ribosomes using a remarkable template-swapping mechanism known as *trans*-translation (Keiler et al., 1996). Acting first as a tRNA, tmRNA is aminoacylated with Ala and delivered to the A site of stalled ribosomes by EFTu. Following transfer of the nascent peptide to Ala-tmRNA, the ribosome releases the non-stop mRNA and resumes translation on a short open reading frame in tmRNA now positioned in the canonical mRNA channel. The tmRNA ORF encodes a 10-amino-acid tag that targets the nascent peptide for proteolysis. Finally, at the tmRNA-encoded stop codon, the tagged peptide is released and the ribosomal subunits are recycled for additional rounds of translation (reviewed in Moore and Sauer, 2007).

In this review, we discuss recent progress in understanding how tmRNA recognizes and enters ribosomes stalled on non-stop mRNAs. To avoid aborting productive protein synthesis, tmRNA must react with stalled ribosomes only. Because this selectivity arises from the way that the tmRNA–SmpB complex interacts with the ribosomal A site, questions of selectivity cannot be separated from questions of how tmRNA and SmpB enter the ribosome. During canonical translation, entry into the ribosome is managed by robust mechanisms that select cognate tRNAs from the cellular pool of aminoacyl-tRNAs. Proper codon–anticodon pairing promotes acceptance and reactivity of cognate tRNA, allowing it to engage these mechanisms efficiently. tmRNA has no anticodon and thus no ability to form codon–anticodon interactions. Yet it is loaded by the canonical factor EFTu, so the tmRNA–SmpB complex must somehow promote GTP hydrolysis on EFTu, and it ultimately undergoes peptidyl transfer, so it must be accommodated into the A site. Recent studies reveal that not only does SmpB stabilize tmRNA in the cell and enhance its aminoacylation (Hanawa-Suetsugu et al., 2002), it also plays the critical role in introducing tmRNA into the A site of stalled ribosomes, and upon translocation to the P site, setting the proper reading frame for the ribosome to resume translation on the tmRNA template (Konno et al., 2007; Watts et al., 2009; Weis et al., 2010).

## THE C-TERMINAL TAIL OF SmpB

**FIGURE 1:**
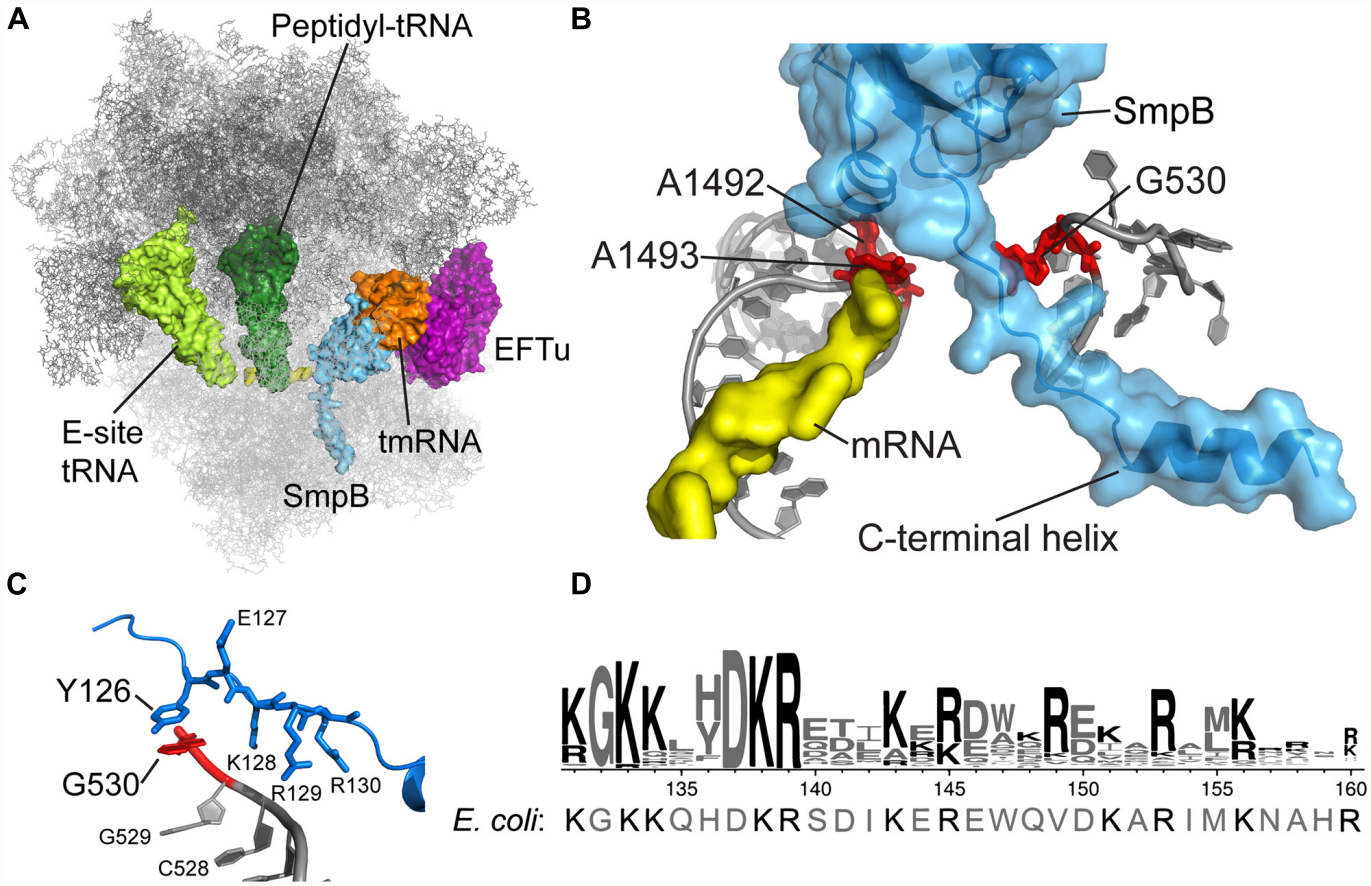
**tmRNA and SmpB binding in the decoding center. (A)** Crystal structure of the *Thermus thermophilus* tmRNA–SmpB complex bound to EFTu on the 70S ribosome, trapped by kirromycin in the pre-accommodation state. Only the tRNA-like domain of tmRNA is included. Rendered using the coordinates from PDB 4ABR and 4ABS (Neubauer et al., 2012). **(B)** SmpB engages 16S rRNA nucleotides A1492, A1493, and G530 in the decoding center. The C-terminal tail extends into the mRNA channel. **(C)** Conserved SmpB residues interact with G530 and nearby nucleotides. *T. thermophilus* Tyr126 corresponds to *E. coli* His136. **(D)** Logo of the C-terminal tail from 470 SmpB proteins (Andersen et al., 2006) with the *E. coli* sequence shown underneath.

Several key functions of SmpB depend on its ∼30 residue C-terminal tail. This sequence is rich in positively charged side chains Arg and Lys and contains several highly conserved stretches of them starting at residue 131 (KGKK) and 137 (DKR) as shown in **Figure 1D**. Mutation of these residues or truncation of the tail abolishes SmpB’s ability to support tmRNA activity *in vivo*, though the resulting SmpB mutants bind to tmRNA efficiently and maintain their affinity for the ribosome (Jacob et al., 2005; Sundermeier et al., 2005). The idea that the C-terminal tail might be involved in promoting entry of tmRNA into stalled ribosomes came from two observations: (1) mutation of the tail blocks early steps in *trans*-translation, prior to peptidyl transfer (Sundermeier et al., 2005), and (2) cryo-EM and chemical probing experiments indicate that it binds near the decoding center in the ribosomal A site (Kaur et al., 2006; Kurita et al., 2007; Nonin-Lecomte et al., 2009). Indeed, the structure of the tRNA-like domain of tmRNA bound to SmpB resembles a canonical tRNA in shape, with SmpB effectively substituting for the anticodon stem-loop (Bessho et al., 2007). The site where the C-terminal tail leaves the body of the protein corresponds to the location of the anticodon in a canonical tRNA.

The crystal structure of the *Thermus thermophilus* tmRNA–SmpB complex bound in the A site of the 70S ribosome confirms these earlier insights and provides a clarifying snapshot of tmRNA and SmpB entering the ribosome (**Figure 1A**; Neubauer et al., 2012). Trapped by kirromycin, the complex represents the pre-accommodation state for tmRNA–SmpB, following GTP hydrolysis but prior to the dissociation of EFTu. The structure effectively puts to rest an earlier controversy regarding how many SmpB molecules are bound to tmRNA: the stoichiometry of tmRNA to SmpB is unambiguously 1:1, consistent with arguments put forward by Karzai (Sundermeier and Karzai, 2007). SmpB is positioned in the decoding center in the 30S subunit, near the conserved nucleotides A1492 and A1493 (**Figure 1B**). The C-terminal tail of SmpB, including residues 133–160 of the *E. coli* protein, lies within the A site and extends into the mRNA channel downstream. Although the tail is unstructured in solution, residues 142–160 form an alpha helix within the mRNA channel, making interactions with 16S rRNA and the S5 protein. Using this structure as a guide, we will discuss the role of the SmpB C-terminal tail in engaging the decoding machinery of the ribosome that is normally used to select cognate tRNAs.

## ENTRY OF tmRNA INTO THE RIBOSOME

Transfer-messenger RNA’s lack of an anticodon raises the question of how it is able to enter the ribosome and react with the nascent peptide. During canonical translation, codon–anticodon pairing promotes the reactivity of cognate tRNAs through well-characterized mechanisms. Cognate tRNAs are selected by two kinetic discrimination steps separated by the hydrolysis of GTP by EFTu (Daviter et al., 2006). After initial binding of the EF-Tu-GTP-tRNA ternary complex to the ribosome, correct base pairing in the codon–anticodon helix promotes conformational changes of conserved 16S rRNA nucleotides A1492, A1493, and G530 (Ogle et al., 2001). These local movements lead to global conformational changes in the ribosome that close the 30S subunit over the tRNA (Ogle et al., 2002), leading to the activation of GTP hydrolysis by EFTu. Cognate tRNAs trigger GTPase activation more rapidly than non-cognate tRNAs through this induced-fit mechanism (Pape et al., 1999; Gromadski and Rodnina, 2004). Following GTP hydrolysis, the second selection step, “proofreading,” occurs as the tRNA is released from EFTu and is fully accommodated into the A site. As in the first selection step, cognate tRNAs undergo more rapid and efficient accommodation than non-cognate tRNAs (Pape et al., 1999).

During *trans*-translation, the decoding center engages not an RNA duplex but instead the C-terminal tail of the SmpB protein. The crystal structure of the *T. thermophilus* tmRNA–SmpB complex bound to the ribosome shows that SmpB engages the decoding center such that A1492 and A1493 flip out of helix 44, as in canonical decoding, albeit in a somewhat different conformation (**Figure 1B**; Neubauer et al., 2012). Equilibrium binding assays further indicate that SmpB binding protects A1492 and A1493 from reacting with chemical probes (Nonin-Lecomte et al., 2009). We tested the hypothesis that SmpB activates EFTu by inducing the same conformational changes in these conserved nucleotides as occurs during canonical decoding. Surprisingly, although mutation of A1492 and A1493 dramatically reduces the rates of both steps of tRNA selection for Phe-tRNA^Phe^, as shown previously (Cochella et al., 2007), these mutations have essentially no effect on EFTu GTPase activation or peptidyl transfer in the context of tmRNA loading (Miller et al., 2011).

## EFTu ACTIVATION BY tmRNA–SmpB

The co-crystal structure of the tmRNA–SmpB complex bound to the 70S ribosome also revealed an unexpected interaction between G530 of the 16S rRNA and the Tyr126 side chain in the SmpB tail (**Figure 1C**). Broadly conserved as aromatic, this residue corresponds to His136 in *E. coli*. By binding G530, His136 may mimic the effect of codon–anticodon pairing on the conformation of this key nucleotide, favoring domain closure and EFTu activation. The importance of this base-stacking interaction was underscored by the finding that the rate of GTP hydrolysis by EFTu was drastically reduced in the His136Ala mutant (Miller and Buskirk, 2014). In contrast, the His136Tyr mutation has only a minor effect, presumably because Tyr is also capable of forming a strong interaction through stacking of its aromatic ring with G530. His136 appears to be the primary or sole determinant in promoting EFTu activation: other SmpB mutations have little or no effect including mutation of residues predicted to contact A1492 and A1493, mutation of the conserved DKR sequence just downstream of His136, and deletion of the last 20 amino acids in the tail.

Further biochemical analysis revealed another surprise: release of tmRNA from EFTu is remarkably facile and can even occur without GTP hydrolysis. With the His136Ala SmpB mutant, peptidyl transfer to Ala-tmRNA occurs at a faster rate than GTP hydrolysis (Miller and Buskirk, 2014). In addition, peptidyl transfer to Ala-tmRNA occurs rapidly even in the presence of kirromycin (Shimizu and Ueda, 2006; Miller and Buskirk, 2014), an antibiotic that blocks release of canonical aminoacyl-tRNAs from EFTu following GTP hydrolysis. These data imply that tmRNA is easily released from EFTu upon ribosome binding and that GTP hydrolysis is not essential to the loading process; it follows that GTPase activation is likely not a determinant for selectivity as it is for canonical decoding. This may be due to the fact that EFTu has a lower binding affinity for Ala-tmRNA than for regular aminoacylated tRNAs (Barends et al., 2000, 2001). Contacts between the large tmRNA molecule and the ribosome may also promote release from EFTu (Shimizu and Ueda, 2006).

## ACCOMMODATION AND PEPTIDYL TRANSFER TO tmRNA

Following release from EFTu, tmRNA is accommodated in the A site where it participates in peptidyl transfer. Interestingly, the relative importance of conserved residues in the SmpB tail is reversed in this step. His136, the key residue in EFTu activation, is not required for efficient peptidyl transfer (Miller and Buskirk, 2014); conversely, several SmpB tail residues that play a minimal part in activation of EFTu are essential for accommodation of tmRNA into the A site. Mutation of the DKR sequence starting at residue 137 abolishes tagging of stalled proteins *in vivo* as well as peptidyl transfer to tmRNA *in vitro* (Sundermeier et al., 2005; Miller et al., 2011). Lys138 and Arg139 form salt bridges with the sugar phosphate backbone of 16S rRNA just upstream of G530 (Neubauer et al., 2012); this interaction may serve as a pivot point for SmpB during accommodation. Likewise, mutation of positively charged residues further downstream in the tail, including Lys143, Arg145, and Arg153, also interferes with tagging activity, as does mutation of Trp147 (Kurita et al., 2010; Miller et al., 2011). Positioned in the mRNA channel, these residues presumably stabilize binding of the tail.

While we have identified key residues essential for promoting peptidyl transfer, we also find that the general helical structure of the C-terminal tail is important. The crystal structure of the tmRNA–SmpB 70S ribosome complex confirmed earlier speculation that the SmpB tail forms an alpha helix within the mRNA channel when bound to the ribosome (Neubauer et al., 2012). Indeed, proline substitutions that disrupt helical structure reduce tagging *in vivo* and peptidyl transfer to tmRNA *in vitro* (Miller et al., 2011). The helical structure of the tail positions the positively charged residues for interactions with the rRNA in the mRNA channel and Trp147 for hydrophobic interactions with the S5 protein (Neubauer et al., 2012). These interactions stabilize binding of the tmRNA–SmpB complex during accommodation in the A site, as the tmRNA–SmpB complex swivels into position (accommodates) to undergo peptidyl transfer. Of course, these interactions cannot be too strong, because they must be broken as the tmRNA–SmpB complex is translocated into the P site. Hydroxyl radical probing and cryo-EM studies show that during translocation, the tail shifts from an extended conformation in the mRNA tunnel to a compact structure folded under the body of SmpB in the 30S P site (Kurita et al., 2007; Weis et al., 2010).

## THE ROLE OF mRNA LENGTH IN SELECTIVITY

The fact that binding of the SmpB tail in the mRNA channel is essential for peptidyl transfer to tmRNA provides a simple model for how tmRNA reacts selectively with non-stop mRNAs. Ribosomes actively translating intact transcripts cannot properly position the SmpB tail to promote accommodation because the mRNA and the tail directly compete for the same binding site. The effect of mRNA length on tmRNA activity was quantitated and it was seen that the efficiency of peptidyl transfer to tmRNA decreases as the length of the mRNA increases. Although the addition of three or six nucleotides downstream of the P site codon has no effect on the k_cat_ for peptidyl transfer, rates are reduced threefold by 9 nt and 10-fold by 12 nt at this position (Ivanova et al., 2004). A length of 12 nt corresponds well with the length of mRNA protected inside the channel: the region 4–7 nt downstream of the P site codon is protected by rRNA, while the region 8–12 nt downstream is protected by the S3, S4, and S5 proteins (Yusupova et al., 2001). The kinetic data show that when mRNA extends increasingly close to the edge of the ribosome, peptidyl transfer to tmRNA is increasingly inhibited.

Conformational dynamics in the mRNA channel may play a role in regulating tmRNA activity: longer mRNAs could stabilize a closed channel conformation, clamping down on the downstream mRNA, while truncated mRNAs could allow a more open conformation. The connection between the shoulder and head of the 30S subunit forming the latch at the opening of the tunnel is clearly dynamic; opening and closing are observed during translocation (Frank and Agrawal, 2000). Perhaps the SmpB tail samples the channel when it is in an open conformation, forming a helix and biasing the channel toward a closed state. The alternative in which the mRNA channel remains closed seems less plausible, as it would require the ∼30-residue tail to bind first in the A site and then snake back down the closed channel. Channel dynamics could explain why the addition of 6–9 nt downstream of the P site codon has only a small effect on rates of peptidyl transfer to tmRNA: an intermediate mRNA length may not adequately stabilize the closed conformation because it fails to adequately engage the S3, S4, and S5 proteins.

Although long mRNAs block binding of the C-terminal tail in the mRNA channel and inhibit peptidyl transfer, EF-Tu activation rates are not affected by mRNA length (Kurita et al., 2014). This implies that rates of initial binding and EF-Tu activation are independent of mRNA length and that GTP is hydrolyzed when tmRNA is delivered to the ribosome, whether or not tmRNA reacts with the nascent peptide and ends up tagging it for destruction. Although this spurious GTP hydrolysis may waste energy, the cost is probably minimal given the fairly low concentration of tmRNA (Moore and Sauer, 2005) and its weak affinity for EF-Tu (Barends et al., 2000, 2001). Taken together, the kinetic studies of tmRNA show that selectivity for truncated mRNA takes place during the accommodation step, when tmRNA and SmpB dissociate from the ribosome if the C-terminal tail cannot bind into the mRNA channel.

Comparison of the mechanism of selection of tmRNA and regular tRNAs highlights the different constraints faced by *trans*-translation and canonical translation. Two selection steps are required during the decoding of regular aminoacyl-tRNAs: initial selection and proofreading (reviewed in Zaher and Green, 2009). While non-cognate tRNAs are efficiently rejected before GTP hydrolysis, some proportion of near-cognate tRNAs make it through and are rejected after GTP hydrolysis. This strategy of multistep discrimination is required to achieve high fidelity because a single selection based on base pairing between the codon and anticodon alone is not sufficient to discriminate between the correct and incorrect substrates. In contrast, two consecutive selection steps may be unnecessary for tmRNA due to the absence of confusingly similar molecules. Selectivity can be achieved in a single step: the accommodation of tmRNA–SmpB into the A site.

## mRNA DECAY DURING RIBOSOME STALLING

The relatively clear picture emerging from the *in vitro* studies is muddied somewhat by the complexity of tmRNA activity *in vivo*. When first describing the *trans*-translation model, Sauer proposed that ribosome stalling on non-stop mRNAs gives tmRNA increased opportunity to enter and recycle these complexes (Keiler et al., 1996). However, later studies seem to suggest that *trans*-translation can also occur at internal sites within intact mRNAs. It was seen that clusters of rare Arg codons, for example, led to pausing and tmRNA action as the ribosome waits for the limiting tRNA^Arg^ that decodes AGA (Roche and Sauer, 1999). Termination at inefficient stop codons also leads to tmRNA-tagging of the nascent peptide (Roche and Sauer, 2001; Collier et al., 2002; Hayes et al., 2002). One solution to these conflicting results is that pausing of translation can lead to degradation of the downstream mRNA, and in some cases cleavage of the mRNA in the ribosomal A site, such that the real target of tmRNA is always shortened (truncated) non-stop mRNA. Such mRNA degradation products have been detected in a variety of contexts (Sunohara et al., 2004; Li et al., 2006, 2008; Garza-Sanchez et al., 2008).

Working out how mRNA downstream of stalled ribosomes is cleaved/processed and how this affects tmRNA activity has proven to be quite challenging. At present, the best characterized example is the appearance of transcripts that are truncated at the A site codon when the ribosome stalls at inefficient stop codons (Hayes and Sauer, 2003). At first, the model was that an endonuclease cleaves the mRNA in the A site and that this was required for robust tmRNA activity. Excellent follow-up work by Hayes and co-workers however, has shown that the story is considerably more complicated. The identity of the enzyme that cleaves the mRNA in the A site remains unknown, and given that the 3′-mRNA fragment has not been detected, it may be that it is not a true endonuclease after all. What we know for certain is that exonucleases play a key role in RNA processing at stalled ribosomes. RNase II exonucleolytically processes transcripts back to ∼15 nt downstream of the P site codon, and without this trimming, further processing of mRNA products back to the A site codon does not occur (Garza-Sanchez et al., 2009). In cells lacking RNase II, even though the amount of mRNA truncated at the A site codon is significantly reduced, the level of tmRNA tagging and the rates of ribosome recycling are not affected (Janssen et al., 2013). These and other studies argue that *in vivo*, robust tmRNA activity occurs on mRNAs processed up to the 3′-boundary, with 15–21 nt of remaining mRNA downstream of the P site codon (Sunohara et al., 2004; Garza-Sanchez et al., 2006; Li et al., 2008). These data are difficult to reconcile with the kinetic data discussed above. Perhaps ribosome pausing, even without mRNA decay, gives tmRNA enough time to enter and recycle the ribosome, even though the rate of peptidyl transfer to tmRNA is 10-fold slower than it would be if the mRNA were truncated in the A site.

## CONCLUDING REMARKS

**FIGURE 2:**
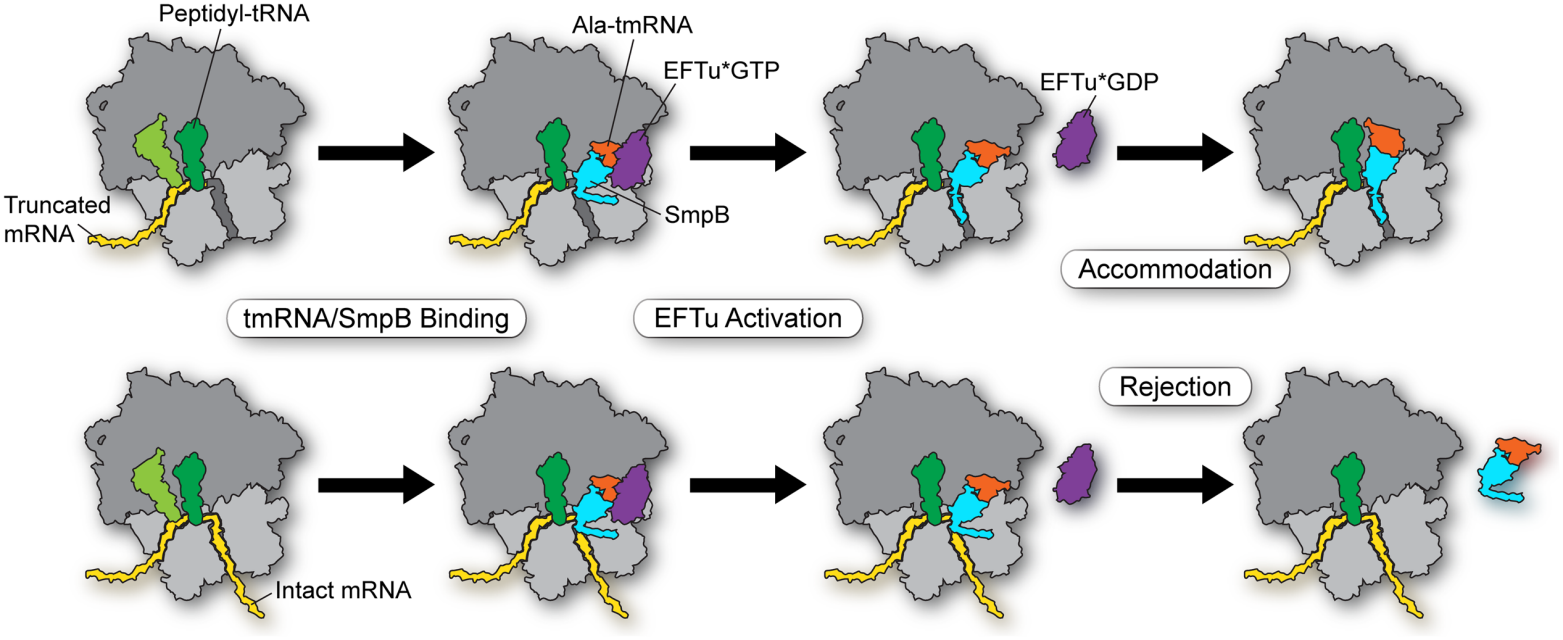
**Model for selectivity in the early steps in *trans*-translation.** EFTu delivers the Ala-tmRNA–SmpB complex to the A site regardless of whether the ribosome sits on a truncated message **(top)** or an intact message **(bottom)**. Stacking of His136 on G530 in the decoding center triggers GTP hydrolysis and release of tmRNA from EFTu. These steps are independent of mRNA length. Binding of the SmpB C-terminal tail within the mRNA channel promotes accommodation of tmRNA into the A site in order to participate in peptidyl transfer **(top)**. Intact mRNAs bias the equilibrium of the mRNA channel toward a closed conformation that blocks binding of the SmpB tail, leading to rejection of the tmRNA–SmpB complex on actively translating ribosomes **(bottom).**

In conclusion, we propose the following model for how tmRNA-SmpB selectively enters stalled ribosomes (**Figure 2**, top) and not actively translating ribosomes (bottom). First, EFTu delivers the Ala-tmRNA-SmpB complex to the A site where binding is stabilized by the body of the SmpB protein with the ribosome. His136 stacks on G530 in the decoding center, triggering GTPase activation (and hydrolysis) and release of tmRNA from EFTu (although it may be that tmRNA is sometimes released prior to GTP hydrolysis). These initial steps occur independent of the mRNA length. But as the tmRNA-SmpB swivels into the A site in order to participate in peptidyl transfer, tmRNA-SmpB dissociates if the C-terminal tail (residues 142–160) is not effectively bound within the mRNA channel, forming a helix. Binding of the tail within the tunnel depends on positively charged residues of SmpB interacting with rRNA and Trp147 interacting with the S5 protein. While it is unclear exactly when proper positioning of the tail must occur in order to promote accommodation, we speculate that the dynamic equilibrium between open and closed forms of the channel is critical, such that intact mRNAs favor a closed conformation that normally blocks tail binding, leading to rejection of the tmRNA-SmpB complex on actively translating ribosomes.

Binding of ribosome rescue factors to the mRNA channel is an effective solution to the problem of identifying non-stop mRNAs. In bacteria, an alternative release factor, ArfB, can enter stalled ribosomes and catalyze hydrolysis of peptidyl-tRNA (Handa et al., 2011) using a GGQ domain common to all class I release factors. Like tmRNA-SmpB, ArfB binds ribosomes independent of a stop codon through a C-terminal tail that forms a helix within the mRNA channel downstream of the A site (Gagnon et al., 2012). This helix has conserved positively charged residues essential for its activity. Ribosomes with intact mRNA are not targeted by ArfB, presumably because the mRNA blocks interactions with the ArfB tail (Handa et al., 2011; Shimizu, 2012).

In eukaryotes, the Dom34/Hbs1 complex rescues stalled ribosomes in an mRNA length dependent manner. The mammalian complex discriminates against transcripts with RNA more than 13 nt downstream of the P-site codon (Pisareva et al., 2011). In yeast, the cutoff is not as sharply defined: subunit splitting occurs rapidly with transcripts shorter than 23 nt after the P site, at intermediate rates between 23–30 nt, and significantly slower on transcripts longer than 30 nt after the P site (Shoemaker and Green, 2011). This length dependence is thought to be mediated by Dom34 binding near the decoding center in the ribosomal A site and by the N-terminal domain of Hbs1 binding near the S3 protein in the mRNA channel (Becker et al., 2011). Structural analyses of the archaeal ortholog of Dom34, known as Pelota (Becker et al., 2012), indicate that it has a similar binding mode as Dom34. In all three domains of life, the machinery that rescues ribosomes selectively reacts with stalled translation complexes by checking for the absence of mRNA using a protein factor to bind the A site and mRNA entry site.

## Conflict of Interest Statement

The authors declare that the research was conducted in the absence of any commercial or financial relationships that could be construed as a potential conflict of interest.
